# Evaluation of root-lesion nematode (*Pratylenchus zeae*) resistance assays for sugarcane accession lines

**DOI:** 10.21307/jofnem-2021-067

**Published:** 2021-07-29

**Authors:** S. A. Bhuiyan, K. Garlick

**Affiliations:** 1 Sugar Research Australia (SRA), 90 Old Cove Road, Woodford, Qld, 4514, Australia; 2 The Queensland Micro- and Nanotechnology Centre, Griffith University, Nathan Campus, Brisbane, Australia

**Keywords:** Root-lesion nematodes, *Pratylenchus zeae*, Sugarcane, Resistance, Screening for resistance

## Abstract

No sugarcane cultivar is resistant to root-lesion (*Pratylenchus zeae*) nematode in Australia. Sugar Research Australia commenced a research project to evaluate new sets of accession lines derived from introgression breeding between wild relatives of sugarcane and commercial parents. No established method of assessment was available for comparing the resistance of sugarcane in the glasshouse. This study aimed to determine the most suitable assessment method for comparing resistance in accession lines. Two resistance parameters were measured, (i) number of nematodes per plant, and (ii) number of nematodes per g of roots. Nine screening trials were conducted from 2011 to 2020. Resistance parameter number of nematodes/plant had less variations within trials compared to nematodes/g roots, although both parameters were equally repeatable. Number of nematodes/g of root were negatively correlated (*r* = ‒0.29 to ‒0.86, *p* ≤ 0.001) with root biomass in all nine trials, and with shoot biomass in three out of seven trials (*r* = ‒0.23 to ‒0.31, *p* ≤ 0.05). In contrast, the number of nematodes per plant were positively correlated with shoot biomass (*r* = 0.25–0.53, *p* ≤ 0.05) in three out of seven trials and with root biomass (*r* = 0.17–0.27, *p* ≤ 00.05) in three out of nine trials. These results clearly indicated that nematodes per g root is related to reduction in root biomass and shoot biomass.

Plant-parasitic nematodes are major pathogens to sugarcane worldwide (Ramouthar and Bhuiyan, 2018). In Australia, they cause 5–20% yield loss/year, costing over $80 million in productivity in Australia (Blair and Stirling, 2007). Lesion nematodes, *Pratylenchus* spp, predominantly *P. zeae*, are the most important nematodes pests of sugarcane in Australia, found in all sugarcane regions, and can cause significant yield loss (Blair and Stirling, 2007; Blair et al., 1999a, b).

Cultural methods such as crop rotations or fallow provide short-term control in plant crop, and nematode populations bounce back to a damaging level within 12 months when sugarcane is replanted (Stirling, 2008; Stirling et al., 2001). Nematicides are relatively expensive, erratic, and only reduce nematode populations for a few months (Blair and Stirling, 2007). No commercial cultivars tested in Australia are resistant to root-lesion nematodes (Stirling et al., 2011).

A collaborative research project between sugarcane breeders from Australia and China in late early 2000s used new sources of resistance by using wild relatives of sugarcane *Erianthus* spp, and *Saccharum spontaneum* to generate new introgression families (Foreman et al., 2007). Some of the accession lines from these crossings were found to be resistant to root-lesion nematodes in a preliminary study (Stirling et al., 2011). In 2011, a research project was commenced by Sugar Research Australia (SRA, formerly BSES) to determine the level of nematode resistance in the progenies derived from crosses between *Erianthus* spp. and *Saccharum spontaneum* lines with commercial sugarcane. The first component of that work was to develop methods for screening large numbers of sugarcane lines against root-lesion nematodes. Initial research by the authors determined trial conditions, suitable potting media, and extraction methods for root-lesion nematodes (Bhuiyan et al., 2014). The other important aspect was to formulate an assessment method for nematode resistance that is reliable and suitable for screening large numbers of progeny lines. The parameter(s) used to measure disease resistance needs be realistic and repeatable.

In Australia, the resistance of sugarcane accession lines is measured by determining the ability of the root-lesion nematode to reproduce in the roots of the test lines. The nematode reproduction is measured by the number of nematodes per plant at the harvest that has been inoculated with a certain number of nematodes at planting (Bhuiyan et al., 2016; Stirling et al., 2011). The relationships of the number of root-lesion nematodes per plant and its impact on root or shoot biomass were poorly understood. Root-lesion nematodes are obligate parasites of sugarcane and their ability to multiply can be limited by the availability of active roots. Measuring shoot and root growth has been used as parameters in comparing accession lines (Stirling et al., 2011). If accession lines with similar root mass have different numbers of nematodes, then the difference is most likely due to resistance. However, if an accession line has a smaller root system, the lower number of nematodes could be due to the limited root biomass available to the nematodes as a food source.

This study examined the relationships of the parameters used to evaluate root-lesion resistance in sugarcane accession lines from 2011 to 2020 in Australia, and determine a suitable method that would provide reliable and repeatable results.

## Materials and methods

### Trial information

A total of 875 sugarcane accession lines were tested against *P. zeae* from 2011 to 2020 in nine trials at SRA Woodford Pathology Farm, Woodford, Queensland (26.9550° S, 152.7780° E) (Table 1). Among these, 722 lines (year 2011–2016) were from introgression populations and 153 lines (year 2017–2020) from Sugar Research Australia (SRA) core breeding germplasm populations. In total, 15 accession lines, including nine from commercial varieties/parents (including two susceptible controls), three introgression lines, and three *Erianathus* spp, were incorporated in multiple trials to obtain data on their resistance to *P. zeae* (Table 2).

**Table 1.** Trial codes, trial year, number of test lines and types of sugarcane population screened for root-lesion nematodes (*Pratylenchus zeae*) for resistance from 2011 to 2020.

Trial code	Year	Total	Remarks
WFLN19-01	2020	42	Core breeding program
Nep18-1	2018	55	Core breeding program
Nep17-1	2017	56	Core breeding program
Nep16-1	2016	60	Introgression population
Nep16-2	2016	86	Introgression population
Nep14-1	2014	157	Introgression population
Nep13-1	2013	153	Introgression population
Nep12-1	2012	148	Introgression population
Nep11-1	2011	118	Introgression population
Total		875	

**Table 2.** List of 15 sugarcane accession lines used in multiple trials with root biomass, and nematode resistance parameters.

Accession	Type	No. of trials^a^	Shoot biomass	Root biomass	Relative nematodes/plant^e^	Relative nematodes/g root
IJ76-333	*Erianthus* sp.^b^	5	15.6ab^d^	46.7ab	89.5f	74.0c
IJ76-370	*Erianthus* sp.	4	10.3b	53.8a	88.0f	67.3c
IJ76-388	*Erianthus* sp.	4	14.9ab	41.3abc	87.2f	72.7c
KQ228	Core	7	17.8ab	32.7abcd	95.3cde	91.2b
Q135^c^	Core	9	16.1ab	25cd	100.6a	101.8a
Q138	Core	6	15.9ab	28.4bcd	99.3abc	98.7ab
Q183	Core	4	15.0ab	24.6bcd	101.1ab	95.9ab
Q200	Core	7	15.5ab	17.4d	96.2bcd	98.3ab
Q208^ac^	Core	9	16.7ab	28.4bcd	99.3ab	98.3ab
Q232	Core	7	21.1a	28.9bcd	94.3de	90.5b
Q240	Core	5	17.9ab	33.6abcd	99.9abc	94.3ab
Q245	Core	6	17.1ab	26.6bcd	99.4abc	97.8ab
QBYC06-30376	Introgression	4	14.6ab	30.9abcd	92.1def	87.5b
QBYC06-30390	Introgression	4	14.2ab	20.3cd	90.4ef	89.6b
QBYN05-20563	Introgression	4	19.2ab	21.6cd	91.9def	90.2b

**Notes:** ^a^Number of trials the accession was included; ^b^Wild relative of sugarcane; ^c^Susceptible controls; ^d^Mean of trials; means followed by same letter(s) in a column are not significant (*p* < 0.05) using Fisher’s protected least significant difference test; ^e^Relativenematodes/plant or g root = *T*/*S* × 100, where *T* is mean nematodes per plant or g root for test accession line, and *S* is mean nematodes per plant or g root for two susceptible controls (Q208 and Q135).

Details of trial procedures and design of experiments were described elsewhere (Bhuiyan and Garlick, 2021; Bhuiyan et al., 2014; Croft et al., 2015). In short, all accession lines were collected from the SRA germplasm collection, situated at Meringa regional station, Queensland. Stalks were cleaned by stripping of leaves and sheaths, and cut into one-budded setts, and hot water treated at 50°C for 30 min to eliminate systemic diseases and placed in a germination chamber in trays with moist vermiculate. Germinated young plants were planted to pots, and transferred to a glasshouse for inoculation. Inoculation was done by applying approximately 2,000 nematodes per pot, and maintained for approximately 12 weeks before harvesting and assessing for resistance on number of nematodes per plant, and number of nematodes per g of roots. Root and shoot biomass were measured for each plant (Bhuiyan et al., 2016, 2019).

Nematodes were extracted from a mixture of root and potting mix using a modified Whitehead tray method (Whitehead and Hemming, 1965). Approximately 250 g of mixture was placed on double-layered Kleenex^®^ tissue paper (Kimberly-Clark Australia Pty., Mission Point, NSW, Australia) on a steel mesh set in a flat tray. The soil and roots were immersed in water and left for 48 hr at 25 ± 1°C. Nematodes were collected on a 38 μm sieve and then the extract was poured into a 30 ml plastic vial. Extracted nematodes samples were stored at 6°C until counting. Counting of nematodes were performed under a compound microscope (10X–40X) using a Peters counting chamber (Chalex Corporation, Wallowa, Oregon, USA) (Peters, 1952) of 1 ml capacity.

### Data analysis

The number of nematodes per plant was recorded after the enumeration, and the number of nematodes per g of roots was estimated from the total number of nematodes present in total root biomass divided by the weight of the root system. Data were analysed by using linear mixed model to all dataset using *Proc Mixed* in SAS version 9.4 (SAS Institute, Cary, NC). For each trial accession lines were treated as fixed effects. Block (replication) and error term (residual) were treated as random effects. The interactions of the fixed effects were included in the model. Degrees of freedom were adjusted using the Kenward–Roger method (Kenward and Roger, 1997) and normality of residuals was tested using ProcUnivariate of SAS. Nematode counting data were ln(*x* + 1)-transformed before analysis. ProcCorr of SAS was used to calculate correlations from mean data between nematode resistance parameters and root or shoot biomass. Corr plot=matrix in SAS was used to calculate the correlation matrix to determine the linear relationship of root and shoot biomass with nematode damage parameters. On the basis of the linearity, ProcReg of SAS was used to determine the relationship between root biomass or shoot biomass and nematodes per g of roots. The percent of coefficient of variation (CV) within each trial for root and shoot biomass were calculated as CV = 100%(*σ*/*µ*), where *σ* and *µ* are the standard deviation and mean, respectively. As for the log-transformed data a formula, $\mathrm{CV}=\;100\%\sqrt{e^{\ln\;{\left(10\right)}^{2\;}\sigma^{2}}‒1}$, where *σ* is the standard deviation of the log-transformed data, was used (Canchola et al., 2017). In total, 15 accession lines, as described earlier, were selected to determine the repeatability among the trials in relation to nematode resistance parameters using Pearson’s correlations (Table 2). Each accession line was included in at least four of the screening trials. SAS *Corr* in *Fisher* command was used to transform the data to normalize the distribution and stabilize the variance (Steel and Torrie, 1960).

## Results

### Repeatability and reliability

The CVs for shoot biomass varied considerably from 17% in WFLN19-01 trial to 34% in Nep11-1 trial (Table 3). Root biomass had the highest variability among the all parameters in each trial, ranged from 31 to 57%. Least CVs were observed in nematode/plant (~3–6%), followed by nematodes/g root.

**Table 3.** Coefficient of variance (%) among shoot biomass, root biomass, number of nematodes per plant and number of nematodes per g of roots in each trial.

Trial name	Shoot biomass	Root biomass	Nematodes/plant	Nematodes/g root
WFLN19-01	17.3	36.1	3.2	7.9
Nep18-1	20.0	30.9	5.4	17.6
Nep17-1	15.9	39.6	3.4	7.9
Nep16-1	–	38.5	4.1	8.5
Nep16-2	–	38.9	3.7	9.0
Nep14-1	25.7	44.1	4.5	8.1
Nep13-1	31.0	34.6	4.3	6.9
Nep12-1	26.6	36.5	5.7	8.7
Nep11-1	34.5	56.9	3.2	5.9

In 15 accession lines, correlation coefficients were based on common accessions tested between two trials. For both parameters, nematodes/plant and eggs/g root, 10 out of 28 possible combinations that include same accessions, correlations among trials were significant (*p* ≤ 0.05) (Table 4). In majority of the trial combinations correlations coefficients were not significant for root or shoot biomass. No differences in shoot biomass were observed among the 15 accessions except for *Erianthus* accession IJ76-370 and core accession Q232 (Table 2). *Erianthus* accession IJ76-370 produced significantly high root biomass compared to introgressions and core accessions. Relative nematodes/plant was significantly (*p* ≤ 0.05) low in three *Erianthus* lines compared to core accession lines, where three introgression lines had significantly low nematode numbers compared to two control accession lines Q208 and Q135.

**Table 4.** Pearson correlation coefficients among 15 accession lines to measure the repeatability among trials in relation to shoot and root biomass, and nematode resistance parameters.

Trial	By Trial	No. accessions common^a^	Shoot biomass	Root biomass	Nematodes/plant	Nematode/g root
Nep11-1	Nep12-1	8	‒0.15	‒0.52	0.45	‒0.01
Nep11-1	Nep13-1	7	0.36	‒0.84*	0.78*	‒0.35
Nep11-1	Nep14-1	8	‒0.17	0.19	0.33	0.38
Nep11-1	Nep16-2	8	–	‒0.24	0.38	‒0.53
Nep11-1	Nep17-1	6	0.56	‒0.69	0.57	‒0.52
Nep11-1	Nep18-1	8	0.51	‒0.11	‒0.1	0.45
Nep11-1	WFLN19-01	8	0.2	‒0.49	‒0.24	‒0.85**
Nep12-1	Nep13-1	9	0.69*	0.56	0.86**	0.79**
Nep12-1	Nep14-1	8	0.63	0.2	0.69	0.78*
Nep12-1	Nep16-2	8	–	0.57	0.75*	0.75*
Nep12-1	Nep17-1	6	0.1	0.56	0.97***	0.95**
Nep12-1	Nep18-1	8	0.52	‒0.5	0.7	0.59
Nep12-1	WFLN19-01	8	0.13	0.86**	0.7	0.77*
Nep13-1	Nep14-1	9	0.28	0.27	0.51	0.51
Nep13-1	Nep16-2	7	–	0.56	0.9**	0.74
Nep13-1	Nep17-1	5	0.12	0.2	0.89*	0.72
Nep13-1	Nep18-1	6	0.25	0	0.42	0.12
Nep13-1	WFLN19-01	6	0.63	0.47	0.45	0.44
Nep14-1	Nep16-2	9	–	0.86**	0.58	0.6
Nep14-1	Nep17-1	6	0.25	0.47	0.47	0.38
Nep14-1	Nep18-1	6	0.8	0.75	0.42	0.56
Nep14-1	WFLN19-01	7	0.13	0.61	0.4	0.01
Nep16-2	Nep17-1	7	–	0.72	0.91**	0.93**
Nep16-2	Nep18-1	8	–	0.3	‒0.02	‒0.1
Nep16-2	WFLN19-01	9	–	0.64	0.38	0.29
Nep17-1	Nep18-1	8	0.68	0.28	0.82*	0.75*
Nep17-1	WFLN19-01	9	0.58	0.81**	0.87**	0.85**
Nep18-1	WFLN19-01	10	0.09	0.16	0.89**	0.71*

**Note:** ^a^Correlation coefficients between two trials are based on the number of common accession lines provided in column 3.

### Correlations of root or shoot biomass with nematode resistance parameters

Three out of seven trials showed moderate (*r* = 0.25–0.53, *p* ≤ 0.05) positive correlations between nematodes per plant and shoot biomass. Three out of nine trials had weak to moderate (*r* = 0.17–0.27, *p* ≤ 0.05) positive correlations and one had moderate negative correlations (*r* = ‒0.36, *p* ≤ 0.05) between nematode per plant and root biomass (Table 5). Nematodes per g of roots were negatively correlated (*r* = ‒0.23 to ‒0.31, *p* ≤ 0.05) with shoot biomass in four out of seven trials. Nematodes/g root were significantly (*p* < 0.001) negatively correlated with root biomass in all trials, and the value of correlations coefficients (*r*) ranged from ‒0.29 to ‒0.86.

**Table 5.** Pearson correlation coefficients to compare the relationship of number of root-lesion nematodes per plant and number of nematodes per g of roots with shoot biomass and root biomass.

	Nematodes/plant		Nematodes/g roots	
Trial name	Shoot biomass^1^	Root biomass	Shoot biomass	Root biomass
WFLN19-01	0.53**	‒0.07 ns	0.21 ns	‒0.86***
Nep18-1	‒0.04 ns	‒0.09 ns	‒0.31*	‒0.60***
Nep17-1	‒0.03 ns	‒0.36*	‒0.07 ns	‒0.81***
Nep16-1	–	‒0.0009 ns	–	‒0.51***
Nep16-2	–	‒0.06 ns	–	‒0.65***
Nep14-1	0.40***	0.17*	‒0.26**	‒0.61***
Nep13-1	0.06 ns	0.13 ns	‒0.27**	‒0.50***
Nep12-1	0.25*	0.24*	0.01 ns	‒0.29**
Nep11-1	‒0.13 ns	0.27*	‒0.23*	‒0.63***

**Notes:** ^1^Significant (*p*-value) *≤ 0.05, **≤ 0.001, ***≤ 0.0001, ns = no significant.

### Relationships of root and shoot biomass with nematode per g of roots

**Figure 1:**
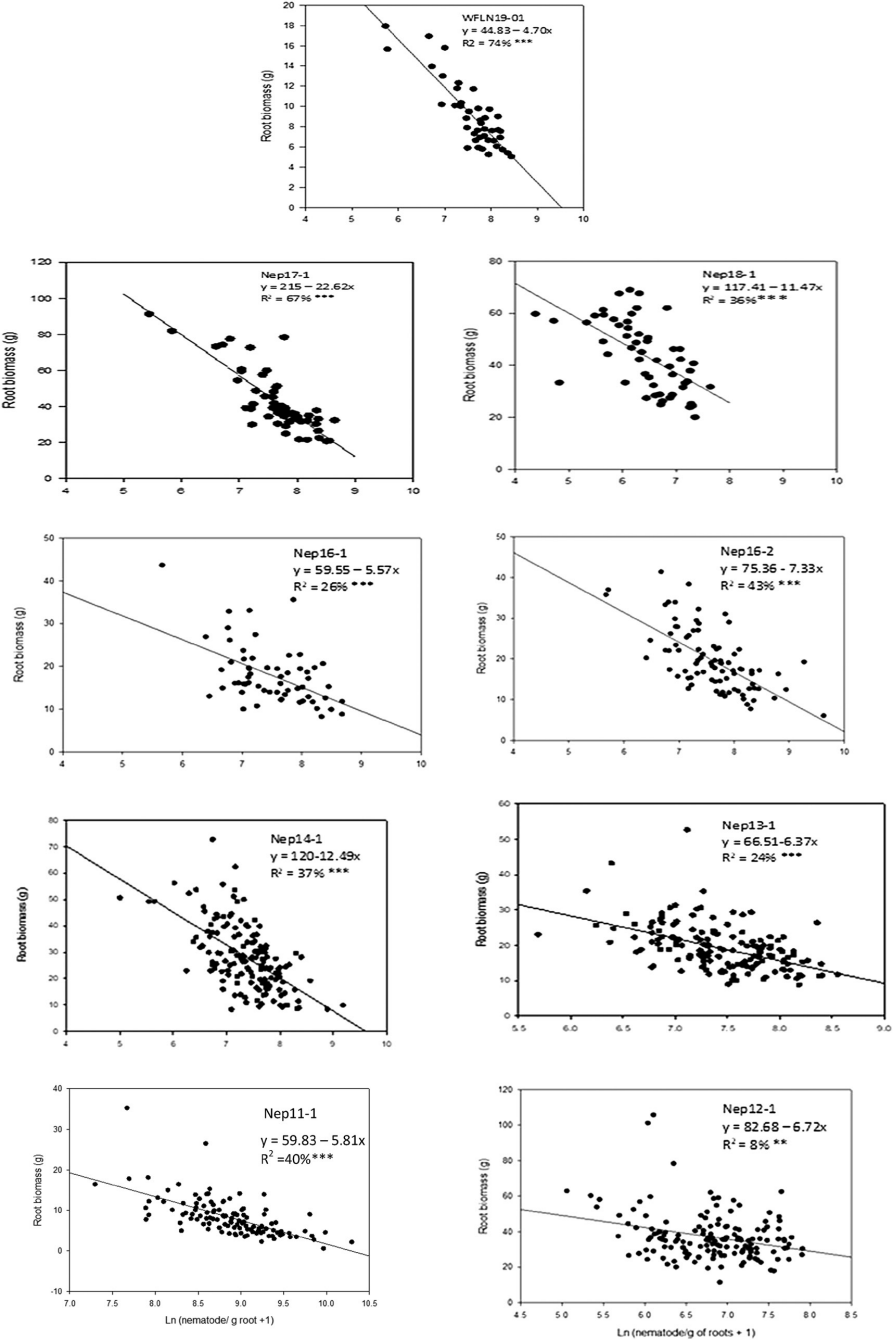
Regressions to show the relationship between root biomass and log (nematodes per g of roots) in nine nematode trials.

**Figure 2:**
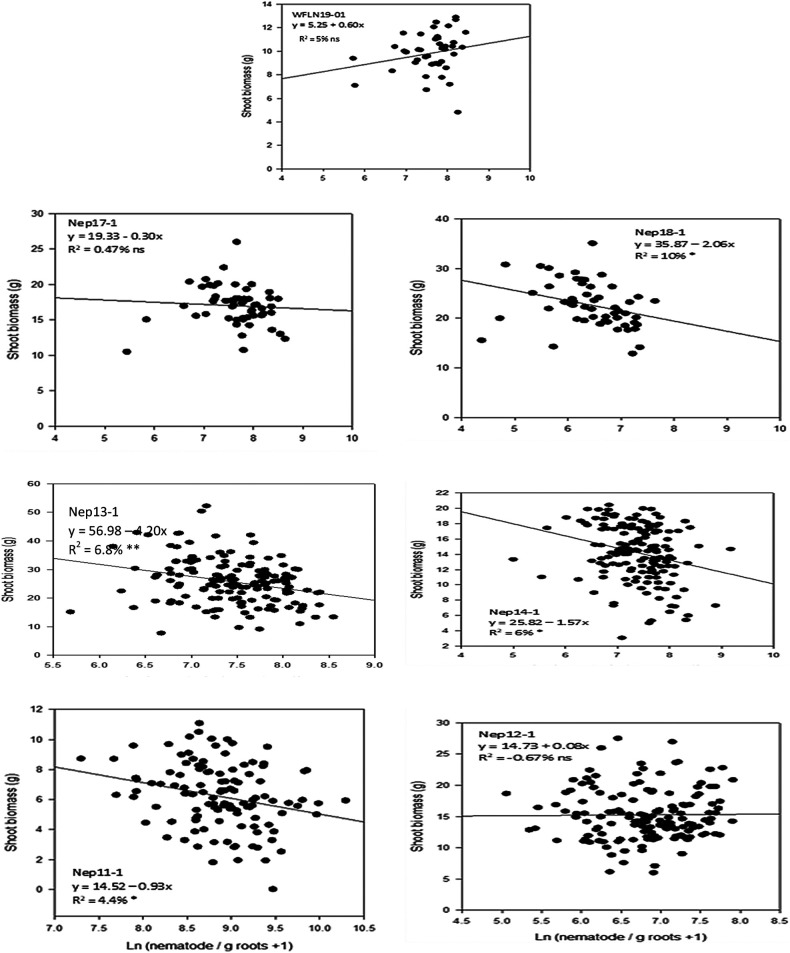
Regressions to show the relationship between shoot biomass and log (nematodes per g of roots) in seven nematode trials.

The relationships between nematodes per g of roots with root biomass were consistently significant (*p* < 0.001) in all trials (Fig. 1). Except for one trial (Nep12-1), nematodes per g of roots explained 25–74% of variations in reduction of root biomass, and both intercepts and slops were highly significant (*p* < 0.0001) in all trials. In trial Nep12-1, nematodes per g of roots were able to explain only 9% of variation in root biomass, although, correlations and regression coefficients were significant (*p* < 0.001). In all trials 8–10% reductions of root biomass were estimated in each unit increase of log-nematode numbers per g of roots. The relationships between nematodes per g roots with shoot biomass varied among trials (Fig. 2). Four out of seven trials had significant (*p* ≤ 0.05) relationships of shoot biomass with nematode per g roots. Although, the regression coefficient of determination only explained 4–8% variations in shoot biomass. Relative nematodes/plant was significantly low in all *Erianthus* lines compared to core accessions, except for introgression lines. On the other hand, nematode/g roots were significantly low *Erianthus* lines compared to rest of the accession lines including introgression lines.

## Discussion

As reliable parameter for assessment for sugarcane accession lines against root-lesion nematodes depends on two important criteria, low variability within a trial and repeatable among trials. Nematode number per plants had slightly lower variabilities (CV) within a trial compared to nematodes/g root, and similar repeatability among the trials.

Lesion nematodes per g of roots on a wide range of sugarcane introgression and commercial breeding lines were negatively correlated with root biomass in all trials and with shoot biomass in four out of the seven trials. In contrast, nematodes per plants had mostly positively or weakly correlated with shoot or root biomass. This is in agreement with our earlier research that implicated high nematode numbers were related to higher root or shoot biomass in sugarcane (Bhuiyan et al., 2016). In most crops the parameter ‘nematodes per plant’ has been used as an indicator for resistance measurement (Bhuiyan et al., 2019; Stirling et al., 2011; Thompson et al., 2009). In sugarcane, this is the first study in sugarcane that compared two resistance indicators to find an appropriate parameter. Our study indicated that the evaluation of sugarcane accession lines based on nematodes/plant provided slightly better parameters compared to nematodes/g root for selection of resistant accessions. Plant breeders evaluate large numbers of accessions each year for nematode resistance. It is important to decide which assessment method is appropriate and provides reliable results without delay. Resistance selection should be based on the ability of an accession line to suppress reproduction and maintain shoot and root biomass. The regression analysis clearly demonstrated that nematodes per g of roots were an alternative indicator of varietal resistance for sugarcane to root-lesion nematodes resistance (Fig. 1).

One of the drawbacks of glasshouse trials, selection based on number of nematodes/plant or g root may not lead to a reduction of yield in some accessions. In earlier glasshouse experiments comparing inoculated and unoculated commercial variety Q208 found that this variety supported highest number of root-knot (*Meloidogyne javanica*) and *P. zeae* in separate experiments, at the same time with high root and shoot biomass, suggesting probable nematode tolerance (Bhuiyan et al., 2016). No information on nematode tolerant accessions, as field trials comparing the performance accessions with and without nematode infestation have not been conducted. Nematodes per plant has been used widely to measure the reproductive capabilities of nematodes on test plants in resistance screening trials (Bhuiyan et al., 2019; Stirling et al., 2011; Thompson et al., 2009). As indicators for resistance, nematode numbers per plant is an effective indicator of separating accession lines due to low trial variations and repeatability. However, nematode per g of roots in identifying resistance among accession lines with variable root biomass also effective. Variations in root biomass is quite common among sugarcane varieties in Australia (Pierre et al., 2019).

The variation for root or shoot biomass would also be affected by types of accession lines included in each trial (Table 2). These phenomena can be observed in the relative number of nematodes/plant or g roots. The wild relative of sugarcane *Erianthus* spp have heavy fibrous root system, and resistant to root lesion nematodes (Bhuiyan et al., 2019). On the other hand, all accession lines from the core including standards were susceptible to root lesion nematodes. Introgression lines, which were progenies from crossings between core accession lines and wild relatives of sugarcane (*Erianthus* spp. Or *Saccharum spontaneum*) had some resistance to lesion nematodes (Bhuiyan et al., 2016, 2019).

Analysis of root-lesion nematode trial data from nine years suggested that number of nematodes per g of sugarcane root had clear impact on root and shoot biomass. Resistance parameter based on nematodes/plant or g roots can be used to reliably select sugarcane accession lines from screening trials. For more advanced lines nematodes/g can be used to select for nematode resistance.
